# Infant mortality across EU health systems: heterogeneity encoding, fixed effects regression, and machine learning in a small macro panel

**DOI:** 10.3389/frhs.2026.1893109

**Published:** 2026-09-16

**Authors:** Ecmel Kisa, Adnan Kisa

**Affiliations:** 1 Department of Technology, Faculty of Health Sciences and Technology, Kristiania University of Applied Sciences, Oslo, Norway; 2 Department of Health and Exercise, Faculty of Health Sciences and Technology, Kristiania University of Applied Sciences, Oslo, Norway

**Keywords:** European Union, fixed effects, heterogeneity encoding, infant mortality, machine learning, panel data, predictive validation

## Abstract

**Introduction:**

The infant mortality rate is widely used in comparative health research, but less is known about how panel regression and machine-learning models perform in small macro-level health datasets when they are evaluated using comparable information and the same out-of-period test period.

**Methods:**

Using a balanced panel of 25 European Union countries observed annually from 2000 to 2022 (575 country-year observations, no missing data) assembled from OECD and World Bank sources, we separated an explanatory analysis from a predictive analysis. The explanatory layer used a two-way fixed effects regression with country-clustered standard errors. The predictive layer trained pooled linear regression, tuned Random Forest, Gradient Boosting, and a Multilayer Perceptron, with and without explicit country information, on 2000–2017 and evaluated them on a held-out 2018–2022 window, alongside two naive benchmarks (persistence and historical country mean) and two prediction-compatible fixed effects specifications. Hyperparameters were tuned by expanding-window temporal cross-validation within the training period, leaving the test window untouched.

**Results:**

Under common out-of-period evaluation, a simple persistence benchmark achieved the strongest predictive performance (R^2^ = 0.74), closely followed by tuned Gradient Boosting and Random Forest with country information (R^2^ = 0.71 and 0.69). Explicit country encoding improved the tree-based models, but adding country information was not enough to improve the linear specifications substantially. The prediction-compatible fixed effects and pooled linear models both produced negative out-of-period R^2^ values. Model rankings varied across evaluation windows: in an alternative pre-pandemic split, tuned nonlinear models outperformed persistence. In the explanatory fixed effects model, GDP per capita and the total fertility rate were significant under conventional country-clustered inference, although neither remained significant under a small-cluster wild-bootstrap sensitivity analysis; the substantive covariate block had a partial R^2^ of 0.366 net of country and year effects.

**Conclusion:**

In this EU infant-mortality panel, much of the predictive signal came from persistent country-specific outcome history. Country information improved the tree-based models, while model flexibility also remained important. The strong within-panel fit of the fixed-effects model did not translate into strong out-of-period prediction.

## 1 Introduction

The infant mortality rate (IMR), defined by the World Health Organization and the World Bank as the number of deaths of children under one year of age per 1,000 live births in a given year, is one of the most widely used indicators of population health and social development (1–3). Because it captures survival during a particularly vulnerable stage of life, IMR reflects broader structural conditions, including healthcare-system performance, economic conditions, and the social policy environment (3, 4). In this manuscript, “infant mortality rate” or “IMR” refers to the specific indicator, whereas “infant mortality” refers to the broader phenomenon. Although infant mortality has declined substantially across most European countries in recent decades (5), progress has not been uniform, and important differences remain between EU member states (5–7). Broader evidence on European health systems also shows persistent differences in health outcomes and system capacity across EU countries, supporting the relevance of comparative EU-level analysis (8, 9).

Three strands of literature are particularly relevant to this study. The first concerns cross-country and panel analyses of the socioeconomic and health-system determinants of infant mortality. National-level studies have long linked IMR to income, income inequality, female education, and public health spending (4). Panel studies have further shown that the relationship between health expenditure and infant mortality may depend on governance quality and financing arrangements rather than reflecting a simple direct association (5, 10). Within the European Union, aggregate panel analyses using country fixed effects have also examined determinants of infant mortality. Tavares (11), for example, reported an inverse association between GDP and infant mortality, while the association with public health expenditure weakened after accounting for country heterogeneity. Fixed effects models are commonly used in this literature because they control for unobserved time-invariant country characteristics and focus estimation on within-country changes over time (5, 11). However, standard linear panel specifications may be restrictive when relationships are nonlinear or when interactions between predictors are important (12).

The second strand concerns the use of machine learning (ML) for infant and neonatal mortality prediction. ML methods allow more flexible relationships between predictors and outcomes and have shown strong predictive performance in several settings. Much of this evidence, however, comes from large individual-level or clinical datasets rather than small country-level panels. XGBoost has achieved an AUC of 0.98 for infant death prediction using United States birth certificate data (12), while neural network models have reported accuracy above 95% in low- and middle-income settings (13). Ensemble methods have also produced very high R^2^ values on large WHO and World Bank datasets (14, 15), and multi-country ML studies have reported AUC-ROC values above 0.81 (7). Survey-based applications have used Random Forest and related learners to predict infant mortality (16, 17), while random survival forests have been applied to identify nonlinear and higher-order interactive effects of socioeconomic conditions on infant survival that may be difficult to capture using conventional parametric models (18). Systematic review evidence also suggests that ML can predict neonatal mortality with good accuracy, although most studies rely on clinical or individual-level records rather than small aggregated macro-panels (19).

The third strand concerns methodological work on explanatory and predictive modeling in small tabular and panel datasets. Recent evidence suggests that ML performance on tabular health data is sensitive to sample size and data structure, and that the gains from more flexible algorithms may diminish when the available sample is relatively small (20, 21). This issue is particularly relevant for macro-level country panels, where the number of observations is constrained by the number of countries and years and where a substantial share of variation may reflect persistent country-specific differences. Under these conditions, algorithms that perform well in large individual-level datasets may not necessarily provide the same advantage when applied to small aggregated panels.

These three areas of research have rarely been examined within the same comparative design. In particular, few studies have assessed how the encoding of country heterogeneity, model flexibility, and temporal validation jointly affect prediction in small cross-national health panels. Comparisons between fixed effects models and ML can also be misleading when the two approaches are given different information or evaluated using different criteria. Fixed effects models can explicitly account for country-specific heterogeneity through country indicators or fixed effects (11, 22), while ML models may be fitted only to observed substantive predictors. Similarly, within-panel explanatory fit and out-of-sample predictive performance answer different methodological questions and should not be interpreted as directly equivalent measures of model performance (23). This study addresses these issues using infant mortality in EU countries from 2000 to 2022 as an applied case. The analysis separates an explanatory fixed effects model from a predictive comparison in which the fitted models use the same balanced analytical sample, are given comparable information where feasible, are tuned using temporal cross-validation, and are evaluated on the same held-out period against naive benchmarks. The aim is not to establish causal effects or to identify a universally superior model, but to examine how country heterogeneity, functional form, and temporal persistence influence explanatory fit and out-of-period prediction in this EU macro panel.

## 2 Methods

### 2.1 Research design

The study uses a country-year panel of EU member states covering 2000 to 2022 and is organized into three analytical layers that are kept deliberately distinct. The explanatory layer estimates a two-way fixed effects regression to characterize within-country associations between the predictors and infant mortality after accounting for time-invariant country characteristics and common annual shocks. The predictive layer trains a set of models on observations from 2000 to 2017 and evaluates them out of period on observations from 2018 to 2022. The predictive models use the same training and test observations, harmonized information sets where feasible, and identically calculated performance metrics, reducing the possibility that differences in evaluation design alone drive the comparison. The robustness layer re-examines the predictive findings using an alternative pre-pandemic split, separate evaluation of pandemic and pre-pandemic years, multiple random seeds for the Multilayer Perceptron, and country-level error analysis. Separating these analytical purposes is important because explanatory fit and out-of-sample predictive performance address different questions and should not be interpreted as directly equivalent measures of model performance (23).

### 2.2 Data sources and study sample

Variables were obtained primarily from the OECD Data Explorer (24), which provides internationally harmonized indicators suitable for cross-country comparison, and were supplemented with World Bank Open Data for selected variables not consistently available from the OECD source (2). The unit of analysis is the country-year. Of the 27 EU member states considered, Cyprus and Malta were excluded before analysis because complete longitudinal data could not be assembled across the variables required for the study. The resulting analytical dataset contains 25 countries observed annually for all 23 years from 2000 to 2022, giving a fully balanced panel of 575 country-year observations. There are no missing values in the variables included in the analysis, and no imputation was therefore required. All explanatory and predictive analyses draw from this same complete 575-observation panel; for the predictive analyses, 450 observations from 2000 to 2017 form the training sample and 125 observations from 2018 to 2022 form the held-out test sample. The 25 included countries and their coverage are listed in Supplementary Table S3, and variable definitions, units, and sources are reported in Table 1.

**Table 1:** Study variables grouped by conceptual dimension, unit, and data source.

Dimension	Variable	Unit	Main source
Outcome	Infant mortality rate	Deaths under age 1 per 1,000 live births	OECD
Economic conditions	GDP per capita	PPP-adjusted USD	OECD
Economic conditions	Health expenditure per capita	PPP-adjusted USD	OECD
Health system capacity	Physicians	Per 1,000 population	OECD
Health system capacity	Nurses	Per 1,000 population	OECD
Health system capacity	Hospital beds	Per 1,000 population	OECD
Preventive and demographic	Total fertility rate	Births per woman	OECD
Preventive and demographic	DTP vaccination coverage	% of one-year-old children immunized	World Bank
Social and behavioral	Female unemployment	% of female labor force	OECD
Social and behavioral	Alcohol consumption	Liters of pure alcohol per capita (15+)	OECD
Social and behavioral	Female tertiary education	Gross enrollment ratio (%)	World Bank

The total fertility rate is expressed as births per woman. Female tertiary education is measured as a gross enrollment ratio and may therefore exceed 100%. OECD data were used as the primary source, while World Bank data were used for selected indicators not consistently available from the OECD source.

### 2.3 Variables

The dependent variable is the infant mortality rate (IMR), expressed as deaths of children under one year of age per 1,000 live births (1). The ten predictors were organized into four conceptual dimensions used only to structure their presentation and interpretation; these dimensions were not entered as separate statistical blocks in any model. Economic conditions comprise GDP per capita and health expenditure per capita. Health-system capacity comprises physicians, nurses, and hospital beds per 1,000 population. Preventive and demographic factors comprise the total fertility rate and DTP vaccination coverage. Social and behavioral conditions comprise female unemployment, alcohol consumption, and female tertiary education. The fertility variable is the total fertility rate (TFR), expressed as births per woman. Variable definitions, units, and sources are shown in Table 1.

### 2.4 Multicollinearity diagnostics

Multicollinearity was examined because correlation among predictors can inflate the variance of regression coefficients and make coefficient estimates less stable, which is particularly relevant to the interpretation of the fixed effects results. Variance inflation factors (VIFs) were therefore used as a diagnostic for the regression-based specifications. Multicollinearity does not produce coefficient variance inflation in tree-based learners in the same way, although correlated predictors can still influence split selection and feature-importance measures.

VIFs were calculated in two forms. Pooled VIFs, based on the untransformed predictors, ranged from 1.22 to 4.36 and were below commonly used rule-of-thumb thresholds of 5 and 10 for identifying potentially problematic multicollinearity, although such thresholds should not be interpreted as rigid decision rules (25, 26). More importantly for the fixed effects analysis, VIFs were also calculated after applying the two-way within transformation to the predictors. These values were all below 1.62, indicating little collinearity in the predictor variation remaining after country and year effects were removed. Both sets of VIFs are reported in Supplementary Table S5. The low within-transformed VIFs suggest that multicollinearity is unlikely to account for the coefficient patterns discussed in Section 3.

### 2.5 Explanatory analysis: Two-way fixed effects

The explanatory analysis used a two-way fixed effects panel regression:

$$\mathrm{IM}R_{\mathrm{it}}=\alpha_{i}+\delta_{t}+X_{\mathrm{it}}\beta +\varepsilon_{\mathrm{it}}$$

where IMRᵢₜ denotes the infant mortality rate in country i and year t; *α*ᵢ represents country fixed effects that absorb time-invariant country characteristics; *δ*ₜ represents year fixed effects that absorb shocks common to all countries in a given year; Xᵢₜ is the vector of ten substantive predictors; and *ε*ᵢₜ is the idiosyncratic error term. The model was estimated by OLS with country and year indicator variables, corresponding to a least-squares dummy-variable (LSDV) implementation of two-way fixed effects. Standard errors were clustered at the country level to account for within-country dependence across repeated annual observations.

Because the full-model R^2^ reflects variation explained by the country and year indicators as well as by the substantive covariates, we also estimated the incremental explanatory contribution of the ten predictors after accounting for the fixed effects. This was summarized using a partial R^2^, calculated as 1−RSSfuₗₗ/RSSFE-only, where RSSfuₗₗ is the residual sum of squares from the model containing the fixed effects and all ten predictors and RSSFE-only is the residual sum of squares from a model containing only country and year fixed effects. A joint *F*-test based on country-clustered inference was additionally used to assess whether the ten substantive covariates were jointly associated with IMR conditional on the fixed effects. The fixed effects estimates are interpreted as adjusted within-country associations and not as causal effects.

### 2.6 Benchmark regression specifications

Two simpler regression specifications were included as reference models. The pooled OLS model regresses IMR on the ten predictors and a numeric time trend without country or year fixed effects. Formally,

$$\mathrm{IM}R_{\mathrm{it}}=\beta_{o}+X_{\mathrm{it}}\beta +\gamma t+\varepsilon_{\mathrm{it}}$$

where Xᵢₜ is the vector of ten predictors, t is the numeric time trend, and *γ* is its coefficient. No country or year indicators are included.

The Cobb-Douglas specification provides a multiplicative log-log benchmark motivated by the health-production-function framework, in which population health outcomes are related to economic and health-system inputs (27, 28). It was estimated by OLS after log-transforming IMR and all ten predictors:

$$\ln(\mathrm{IM}R_{\mathrm{it}})=\beta_{0}+\Sigma_{k\;=\;1}1^{0}\;\times \beta_{k}\;\times \ln(X_{k,\mathrm{it}})\;+\varepsilon_{\mathrm{it}}$$

All variables in the analytical panel are strictly positive, so the logarithmic transformation is defined for every observation. In this specification, each *β*ₖ represents the estimated elasticity of IMR with respect to predictor k, conditional on the other included predictors. These coefficients are interpreted as associational rather than causal elasticities. Both benchmark models were trained on observations from 2000 to 2017 and evaluated out of period on the held-out 2018–2022 observations.

### 2.7 Machine learning models

Three machine-learning models were implemented in Python using scikit-learn: Random Forest, Gradient Boosting, and a Multilayer Perceptron (MLP). Random Forest constructs multiple regression trees from bootstrap samples while randomly selecting subsets of predictors at each split, and averages the resulting tree predictions. This procedure reduces variance relative to a single decision tree and can improve out-of-sample generalization (29). Gradient Boosting builds regression trees sequentially by minimizing a specified differentiable loss through functional gradient descent. Under the squared-error loss used in this study, each successive tree is fitted to the residuals from the current ensemble, which represents the relevant special case rather than a universal definition of gradient boosting (30). The MLP is a feedforward neural network composed of fully connected layers whose parameters are estimated by gradient-based minimization of squared-error loss using the Adam optimizer, with L2 regularization applied to the network weights (26). All three models can represent nonlinear relationships and interactions without requiring these relationships to be specified in advance (26, 29, 30).

For Random Forest, the predicted value is the average of B regression trees:

$$\hat{f}(x)=(1/B)\;\Sigma_{\beta =\;1}B\cdot T_{\beta }(x)$$

where Tb(x) denotes the prediction from the b-th tree. Gradient Boosting constructs the prediction function additively:

$$F_{m}(x)=F_{m\text{-}1}(x)+\eta h_{m}(x)$$

where hₘ(x) is the regression tree added at iteration m and *η* is the learning rate. For the MLP, the network maps the input vector through one or more hidden layers using nonlinear activation functions, with model parameters estimated by minimizing the squared-error objective subject to L2 regularization. Hyperparameters for all three machine-learning models were selected using temporally ordered cross-validation within the 2000–2017 training period, while the 2018–2022 test period remained untouched until final evaluation.

### 2.8 Information-set comparison and preprocessing

To distinguish the contribution of functional flexibility from that of country-level heterogeneity, each machine-learning model was estimated under two information sets. The predictor-only specification included the ten substantive predictors and a numeric time trend, defined as calendar year minus 2000, but did not include country identity. The country-informed specification additionally included one-hot encoded country indicators. The two specifications therefore contained the same substantive predictors and the same time trend and differed only in whether country identity was explicitly represented. A numeric time trend was used rather than one-hot encoded year indicators because the 2018–2022 test years were not observed in the 2000–2017 training period. Separate indicator categories for these future years could therefore not be estimated from the training data, whereas the numeric trend provides a defined representation for unseen years.

All preprocessing steps were fitted using training data only or, during hyperparameter tuning, separately within each training fold to prevent information leakage. For pooled linear regression and the MLP, the ten continuous predictors and the numeric time trend were standardized using StandardScaler. The scaling parameters were estimated from the training data and then applied to the test data. Random Forest and Gradient Boosting were fitted using unscaled numeric predictors and the unscaled time trend because tree-based models do not require feature scaling. The fixed effects regression was also estimated using predictors in their original units to preserve the interpretation of the regression coefficients. Where country information was included in the machine-learning models, country identity was represented using one-hot encoding fitted on the training data. All 25 countries were represented in both the training and test periods, so no previously unseen country categories occurred during test-set evaluation. The Cobb-Douglas specification used log-transformed IMR and predictor values. The transformation was defined for all observations because all variables included in that specification were strictly positive.

### 2.9 Naive benchmarks

Two naive benchmarks were included to provide simple reference points for predictive performance. The persistence benchmark uses each country's 2017 infant mortality rate as the prediction for all observations from 2018 to 2022. The historical country-mean benchmark uses each country's average infant mortality rate over 2000–2017 as the prediction for the same test period. Both benchmarks were evaluated on the 2018–2022 test window using the same performance metrics as the fitted models. These benchmarks provide a simple way to assess how much predictive information is contained in recent country-specific outcome history and longer-term country averages without using the macroeconomic and health-system predictors.

### 2.10 Prediction-compatible fixed effects

The two-way fixed effects model is primarily intended to estimate within-country associations rather than to generate forecasts, because year effects for 2018–2022 cannot be estimated from a model fitted only on data from 2000 to 2017. To allow a fair out-of-period comparison with the predictive models, two prediction-compatible fixed effects specifications were therefore used. The first specification estimates the two-way fixed effects model on 2000–2017 and applies a last-observed-year-effect rule, in which the estimated common year effect for 2017 is carried forward to the 2018–2022 test period. This provides a defined forecasting rule without estimating year effects that are not observed in the training data. The second specification replaces unrestricted year fixed effects with a country fixed effect and a linear time trend, allowing the time component to extend into the test period in a defined way. Both specifications were evaluated on the same 2018–2022 test observations and with the same performance metrics as the other predictive models. The original two-way fixed effects model with unrestricted country and year effects was retained for explanatory analysis only and was not used as a forecasting model.

### 2.11 Hyperparameter tuning and evaluation

Hyperparameters for Random Forest, Gradient Boosting, and the MLP were tuned using expanding-window, or rolling-origin, temporal cross-validation entirely within the 2000–2017 training period. The 2018–2022 test period was kept separate and was not used during model selection. This type of temporally ordered validation is appropriate when observations are structured over time because it preserves the chronological order between training and validation data (26). For Random Forest, the search covered the number of trees, maximum tree depth, minimum leaf size, and the number of features considered at each split. For Gradient Boosting, the search covered the number of estimators, learning rate, maximum tree depth, and subsampling fraction. For the MLP, the search covered hidden-layer architecture, L2 regularization strength, and initial learning rate, with scaling carried out within the cross-validation pipeline. The search spaces, selected configurations, and cross-validation results are reported in Supplementary Table S6.

Because the MLP is stochastic and can be sensitive to initialization in small samples, the tuned country-informed MLP was refitted across ten random seeds. The resulting distribution of test performance is reported in Section 3.6. Final predictive performance for all models was evaluated on the held-out 2018–2022 test set using root mean squared error (RMSE), mean absolute error (MAE), and the coefficient of determination (R^2^), calculated in the same way for every model. Because test-set R^2^ is measured relative to prediction by the test-set mean, it can take negative values when a model performs worse than that reference prediction (26). RMSE and MAE are therefore emphasized as the primary measures for comparing predictive error across models.

## 3 Results

### 3.1 Sample and descriptive overview

The final analytical panel comprised 575 country-year observations from 25 EU countries over the period 2000–2022. The panel was balanced and complete, with no missing values in the variables included in the analysis (Supplementary Tables S3 and S4). The mean infant mortality rate was 4.39 deaths per 1,000 live births, with a median of 3.70 and a range from 1.40 to 18.60. The distribution was right-skewed, with substantial cross-country variation in economic conditions, health-system capacity, and social indicators. All analyses drew from the same complete 575-observation panel. For the predictive analysis, all fitted models used the same 2000–2017 training period and were evaluated on the same 2018–2022 test period. This ensured that differences in predictive performance were not attributable to differences in the observations available to the models.

### 3.2 Comparable out-of-period predictive performance

Table 2 reports out-of-period predictive performance on the 2018–2022 test period using the same test observations and the same performance metrics across models. The ranking differed markedly from that suggested by within-panel model fit. The persistence benchmark achieved the strongest predictive performance, with an RMSE of 0.534, an MAE of 0.382, and an R^2^ of 0.737. Among the fitted models, tuned Gradient Boosting with country information performed best, with an RMSE of 0.559, an MAE of 0.428, and an R^2^ of 0.711, followed by tuned Random Forest with country information, with an RMSE of 0.578, an MAE of 0.470, and an R^2^ of 0.691.

**Table 2:** Out-of-period predictive performance on the 2018–2022 test period.

Model	Country information	RMSE	MAE	R²
Persistence benchmark	Country-specific 2017 IMR	0.534	0.382	0.737
Gradient Boosting + Country (tuned)	One-hot encoded	0.559	0.428	0.711
Random Forest + Country (tuned)	One-hot encoded	0.578	0.470	0.691
Random Forest (tuned)	No	0.631	0.504	0.632
MLP (tuned)	No	0.679	0.530	0.574
Gradient Boosting (tuned)	No	0.700	0.536	0.547
MLP + Country (tuned)	One-hot encoded	0.761	0.645	0.464
Cobb-Douglas	No	1.067	0.841	−0.053
Two-way FE OOS	Country fixed effects	1.333	0.979	−0.645
Country FE + linear trend	Country fixed effects	1.442	1.120	−0.924
Linear Regression	No	1.835	1.499	−2.114
Historical country-mean benchmark	Country-specific training mean	2.094	1.511	−3.055

All fitted models were trained on observations from 2000 to 2017 and evaluated on the held-out 2018–2022 period. The two naive benchmarks and the prediction-compatible fixed effects specifications use the rules defined in Sections 2.9 and 2.10. RMSE and MAE are emphasized as the primary measures of predictive error. Test-set R² is calculated relative to prediction by the test-set mean and may therefore be negative. The test period includes the COVID-19 pandemic years, which are examined separately in Section 3.5.

The prediction-compatible fixed effects specifications performed poorly out of period. The two-way fixed effects model using the last-observed-year-effect rule produced an R^2^ of −0.645, while the country fixed effects model with a linear time trend produced an R^2^ of −0.924. Pooled linear regression also performed poorly, with an R^2^ of −2.114, as did the historical country-mean benchmark, with an R^2^ of −3.055. The Cobb-Douglas benchmark produced an R^2^ of −0.053.

When evaluated on the same out-of-period observations, the prediction-compatible fixed effects specifications did not outperform the tuned tree-based models and produced substantially larger prediction errors.

### 3.3 Heterogeneity encoding and functional flexibility

To examine the contribution of country information separately from functional form, Table 3 presents a two-by-two comparison crossing linear versus flexible nonlinear specifications with the absence or presence of explicit country information. Random Forest was used as the representative flexible nonlinear model. Adding country information improved predictive performance in both specifications. In the linear comparison, R^2^ increased from −2.114 to −0.924 and RMSE decreased from 1.835 to 1.442. For Random Forest, R^2^ increased from 0.632 to 0.691 and RMSE decreased from 0.631 to 0.578. Gradient Boosting showed a larger improvement with country information, with R^2^ increasing from 0.547 to 0.711.

**Table 3:** Two-by-two comparison of functional form and country heterogeneity encoding on the 2018–2022 test period.

Functional form	Country heterogeneity	RMSE	MAE	R²
Linear	Not explicitly encoded	1.835	1.499	−2.114
Linear	Explicitly encoded	1.442	1.120	−0.924
Flexible nonlinear	Not explicitly encoded	0.631	0.504	0.632
Flexible nonlinear	Explicitly encoded	0.578	0.470	0.691

The flexible nonlinear rows correspond to the tuned Random Forest specifications. Gradient Boosting showed a larger improvement after country information was added, with R² increasing from 0.547 to 0.711. The linear rows correspond to pooled linear regression with a numeric time trend and the country fixed effects specification with a linear time trend.

The MLP showed a different pattern. Its test R^2^ decreased from 0.574 without country information to 0.464 when one-hot encoded country indicators were included. The country-informed MLP also showed variability across random initializations. Across ten random seeds, mean test R^2^ was 0.467 (SD = 0.106), with values ranging from 0.227 to 0.595. The full stability results are reported in Section 3.6.

### 3.4 Explanatory fixed effects results

The explanatory fixed effects results are reported separately from the predictive comparison. The full two-way FE/LSDV specification produced a full-model R^2^ of 0.882, compared with 0.531 for pooled OLS estimated on the same sample. A model containing country and year fixed effects but no substantive covariates already produced an R^2^ of 0.814. The partial R^2^ of the ten substantive covariates, conditional on the country and year fixed effects, was 0.366. The covariate block was jointly significant under conventional country-clustered inference (F = 3.212, *p* = 0.009). The full-model R^2^ and adjusted R^2^ of 0.882 and 0.869 are therefore reported as measures of fit for the complete specification rather than as measures of the explanatory contribution of the substantive covariates alone.

Using country-clustered standard errors, GDP per capita (*β* = −0.000073, *p* = 0.037) and the total fertility rate (*β* = −3.497, *p* = 0.029) were statistically significant at the 5% level. Nurses per 1,000 population (*p* = 0.065) and DTP vaccination coverage (*p* = 0.087) did not reach the 5% significance threshold, while health expenditure per capita was also not statistically significant (*p* = 0.260). Full coefficients, country-clustered standard errors, and confidence intervals are reported in Table 4 and Supplementary Table S1.

**Table 4:** Two-way fixed effects coefficients with country-clustered standard errors.

Variable	Coefficient	Clustered SE	z statistic	*p* value	95% CI
GDP per capita	−7.31e-5	3.51e-5	-2.08	0.037	[−0.00014, −0.00000]
Health expenditure per capita	3.80e-4	3.37e-4	1.13	0.260	[−0.00028, 0.001]
Physicians per 1,000	−0.2778	0.3037	−0.91	0.360	[−0.873, 0.317]
Nurses per 1,000	0.1631	0.0885	1.84	0.065	[−0.010, 0.337]
Hospital beds per 1,000	−0.0464	0.2427	−0.19	0.848	[−0.522, 0.429]
Total fertility rate	−3.4972	1.6058	−2.18	0.029	[−6.645, −0.350]
Female unemployment	−0.0206	0.0380	−0.54	0.587	[−0.095, 0.054]
Alcohol consumption	−0.1393	0.3482	−0.40	0.689	[−0.822, 0.543]
DTP vaccination coverage	0.1327	0.0776	1.71	0.087	[−0.019, 0.285]
Female tertiary education	−0.0011	0.0103	−0.11	0.912	[−0.021, 0.019]

Coefficients represent adjusted within-country associations after accounting for country and year fixed effects. Standard errors are clustered by country. Country and year indicator coefficients are omitted. Full-model R² = 0.882. Partial R² of the ten substantive covariates conditional on country and year fixed effects = 0.366. Joint F = 3.212, *p* = 0.009 under conventional country-clustered inference.

Two additional analyses were conducted to assess the fixed effects results. First, a nested comparison using the identical analytical sample showed that pooled OLS produced an R^2^ of 0.531, adding country indicators increased R^2^ to 0.863, adding year indicators alone increased it to 0.552, and including both country and year indicators produced an R^2^ of 0.882. Thus, most of the increase in model fit relative to pooled OLS was associated with the inclusion of country indicators rather than year indicators (Supplementary Table S1). Second, because the panel contains only 25 country clusters, the two coefficients that were significant under conventional clustered inference were re-examined using a wild-cluster bootstrap with Rademacher weights, a restricted null, and 9,999 replications. The resulting *p*-values were 0.156 for GDP per capita and 0.095 for the total fertility rate. Neither association therefore remained statistically significant under the small-cluster bootstrap sensitivity analysis, and these findings are interpreted cautiously.

A one-year lagged-predictor sensitivity analysis was also conducted to assess the temporal stability of the coefficient estimates. This analysis is interpreted as a sign-stability check rather than as a method for resolving endogeneity or establishing causal effects. All ten coefficients retained the same direction after one-year lagging (Supplementary Table S2). GDP per capita remained negative (lagged *β* = −0.000074, *p* = 0.036) and the total fertility rate remained negative (lagged *β* = −3.272, *p* = 0.031) under conventional country-clustered inference. The positive coefficients for health expenditure, nursing density, and vaccination also retained their direction but remained statistically non-significant or borderline. The lagged analysis therefore indicates that the direction of the estimated associations was stable, while the evidence for the positive health-system coefficients remained limited.

### 3.5 Temporal robustness

Because the primary 2018–2022 test period includes the COVID−19 pandemic, two robustness analyses were conducted. First, the test period was divided into a pre-pandemic segment (2018–2019) and a pandemic-affected segment (2020–2022) using the same models trained on 2000–2017. In 2018–2019, the persistence benchmark had the highest R^2^ at 0.806. During 2020–2022, tuned Gradient Boosting with country information had an R^2^ of 0.687, compared with 0.678 for the persistence benchmark. The country-informed MLP had an R^2^ of 0.245 during 2020–2022. Per-period and per-year results for the primary split are reported in Supplementary Table S7.

Second, the full predictive pipeline, including hyperparameter tuning, was re-estimated using an earlier pre-pandemic split. Models were trained on 2000–2014 and evaluated on the held-out 2015–2017 period. In this analysis, the country-informed MLP had the highest R^2^ at 0.871, followed by Gradient Boosting with country information at 0.860, Random Forest with country information at 0.828, Random Forest without country information at 0.815, and the persistence benchmark at 0.802. The linear model with a time trend produced an R^2^ of −1.067 (Table 5).

**Table 5:** Alternative pre-pandemic split: models re-estimated on 2000–2014 and evaluated on 2015–2017.

Model	RMSE	MAE	R²
Persistence benchmark	0.537	0.395	0.802
Linear + time trend	1.734	1.524	−1.067
RF + time trend (tuned)	0.519	0.415	0.815
RF + Country + time trend (tuned)	0.500	0.386	0.828
GB + Country + time trend (tuned)	0.452	0.342	0.860
MLP + Country + time trend (tuned)	0.432	0.307	0.871

Hyperparameter tuning was repeated entirely within the 2000–2014 training period, and the 2015–2017 test period remained untouched until final evaluation. Per-period and per-year results for the primary split are reported in Supplementary Table S7.

### 3.6 Stochastic stability of the MLP

To assess stochastic stability, the tuned country-informed MLP was refitted across ten random seeds using the same architecture selected by temporal cross-validation and the same 2018–2022 test period. Test performance varied across seeds, with a mean R^2^ of 0.467 (SD = 0.106, range = 0.227–0.595) and a mean RMSE of 0.756 (SD = 0.073) (Supplementary Table S8). The seed-42 result reported in Table 2 had an R^2^ of 0.464. Results for all ten random seeds are reported in Supplementary Table S8.

### 3.7 Country-level prediction errors

Predictions for every test-period country-year for the principal models, together with signed and absolute prediction errors, are reported in Supplementary Table S9. Country-level MAE and RMSE values are reported in Supplementary Table S10. Prediction errors varied across countries and models, and no single model produced the lowest error for every country. The country-level results therefore show variation in predictive accuracy that is not fully reflected in the aggregate test-set metrics.

## 4 Discussion

### 4.1 Principal findings

This study compared explanatory panel regression and out-of-period prediction in a small EU infant-mortality panel after placing the predictive models on a more comparable footing. All predictive models were based on the same complete analytical panel, used the same training and test periods, were evaluated with the same performance metrics, and, where feasible, were given comparable information about time and country identity. Hyperparameters for the machine-learning models were selected using temporal cross-validation rather than the final test period.

The revised analysis yields four main findings that together clarify how model specification, heterogeneity encoding, and temporal structure shaped both explanatory fit and out-of-period prediction in this EU panel. First, the ranking of models under out-of-period prediction differed substantially from the ranking suggested by within-panel fit. In the primary 2018–2022 test period, the persistence benchmark produced the lowest RMSE and MAE and the highest R^2^. Among the fitted models, Gradient Boosting and Random Forest performed best when country identity was explicitly included. By contrast, prediction-compatible fixed effects specifications and pooled linear regression performed poorly out of period. This distinction is important because explanatory fit and predictive performance address different questions and should not be interpreted as interchangeable measures of model quality (23). Second, explicit country information improved both tree-based models. Random Forest improved from an R^2^ of 0.632 to 0.691 after country indicators were added, while Gradient Boosting improved from 0.547 to 0.711. The same improvement was not observed for the MLP, and adding country information did not make the linear specifications competitive with the nonlinear models. These findings indicate that country identity contained predictive information beyond the observed macroeconomic and health-system indicators, but that the way this information was used depended on model form. Third, the explanatory fixed effects results were considerably more cautious than the full-model R^2^ of 0.882 might suggest. Country and year fixed effects alone accounted for a large share of panel variation, while the ten substantive covariates had a partial R^2^ of 0.366 after those fixed effects were accounted for. GDP per capita and total fertility rate were statistically significant under conventional country-clustered inference, but neither remained significant under the wild-cluster bootstrap sensitivity analysis. The lagged-predictor analysis showed stable coefficient directions, but it did not materially strengthen the evidence for the counterintuitive positive coefficients for health expenditure, nursing density, or vaccination. The explanatory results should therefore be interpreted as adjusted within-country associations rather than causal effects. Fourth, model rankings varied across evaluation periods. Persistence was strongest in the primary 2018–2022 window, whereas the tuned nonlinear country-informed models outperformed persistence in the alternative 2015–2017 test period. This temporal variation argues against a general claim that one model family is consistently superior in this setting.

The strong performance of the persistence benchmark is particularly informative. Carrying each country's 2017 infant mortality rate forward produced better predictions in the primary test period than any fitted model based on the selected macroeconomic and health-system indicators. By contrast, the historical country-mean benchmark performed very poorly (R^2^ = −3.055). The difference between these two simple benchmarks suggests that recent country-specific IMR levels contained considerably more predictive information for the following years than a country's longer-term historical average.

### 4.2 Interpretation and relation to prior literature

The explanatory findings are broadly consistent with the cross-country literature showing that infant mortality is related to economic and health-system conditions but that estimated associations are sensitive to the way country heterogeneity is handled. Previous studies have linked infant mortality to income, education, health expenditure, and broader institutional conditions (4, 5, 10). Within Europe, Tavares (11) reported an inverse relationship between GDP and infant mortality while finding weaker evidence for public health expenditure after country-level heterogeneity was taken into account. The negative GDP coefficient in the present fixed-effects analysis is consistent with this literature, although its statistical significance was not robust to the small-cluster bootstrap.

The results also reinforce the importance of distinguishing cross-sectional differences from within-country associations. Avendano (22) showed that the relationship between income inequality and infant mortality changed substantially after country fixed effects were introduced. A similar pattern is visible here. Pooled OLS produced an R^2^ of 0.531, while adding country indicators increased R^2^ to 0.863. Adding year indicators alone produced a much smaller increase, to 0.552. This suggests that persistent differences between countries account for a large part of the variation in infant mortality observed in the panel. It also helps explain why models that explicitly represented country identity performed differently from models relying only on the substantive predictors.

The weak and statistically uncertain coefficients for health expenditure and health-system capacity also fit with previous evidence that greater resources do not translate mechanically into lower mortality. Onofrei et al. (5), Kiross et al. (10), and Anwar et al. (31) emphasize that the relationship between health expenditure and population health depends on the institutional and financing context in which resources are used. In the present study, health expenditure, nursing density, and vaccination coverage did not show robust within-country associations after country and year effects were absorbed and country-level dependence was taken into account. These findings do not imply that these inputs are unimportant for infant health. Rather, aggregate national indicators may not capture differences in allocation, access, quality, efficiency, or the populations reached by health services.

The predictive findings differ from much of the published ML literature on infant and neonatal mortality, where very high levels of predictive performance have often been reported. Studies using large individual-level, survey, or administrative datasets have demonstrated strong performance for Random Forest, boosting methods, neural networks, and related approaches (7, 12, 16, 17). Aizawa (18) also showed that tree-based survival methods can capture nonlinear and interactive socioeconomic patterns that may be difficult to represent using conventional parametric models.

The present study differs from much of that literature in two important respects. The unit of observation is the country-year rather than the individual, and the available training sample is small. The models therefore have far less independent information from which to learn complex relationships. Evidence from tabular prediction research indicates that the relative advantage of flexible ML methods depends strongly on sample size and data structure (20, 21). The results here are consistent with that broader evidence. Tuned tree-based models performed well and benefited from country information, but their advantage over a simple persistence benchmark was limited in the primary test period. The MLP was also sensitive to random initialization, with substantial variation in test performance across seeds, illustrating the difficulties of fitting higher-capacity models in a small macro-level dataset.

The fixed effects forecasting results should be interpreted in a different way. Their poor out-of-period performance does not imply that fixed effects are inappropriate for explanatory panel analysis. Two-way fixed effects models are designed to estimate associations after removing time-invariant country characteristics and common year shocks. Unrestricted future year effects, however, cannot be estimated before those years are observed. The poor predictive performance of the two prediction-compatible FE variants therefore highlights the distinction between a model that explains observed within-panel variation and a model designed to predict future observations. This distinction is central to the separation between explanatory and predictive modeling emphasized by Shmueli (23).

The temporal robustness analysis adds a further qualification. The primary test period includes the COVID-19 pandemic, which created an unusual mortality environment, but the findings cannot be attributed solely to that period. When the entire pipeline was re-estimated using 2000–2014 for training and 2015–2017 for testing, the tuned nonlinear models outperformed persistence. Model rankings therefore depended on the evaluation period, reinforcing the importance of temporal validation rather than drawing conclusions from a single train-test split.

### 4.3 Implications for comparative health services research

The findings have several implications for comparative health services research. First, model evaluation should be aligned with the research objective. Fixed effects models remain useful when the aim is to examine adjusted within-country associations, but their in-panel fit should not be interpreted as evidence of forecasting performance. Conversely, predictive models should be judged using observations that were not used for estimation or tuning.

Second, predictive studies using country-level health data should consider how national heterogeneity is represented. In this study, explicit country indicators improved Random Forest and Gradient Boosting, indicating that observable macroeconomic and health-system variables did not capture all persistent country-level differences relevant to infant mortality. At the same time, country information alone was not sufficient to make the linear specifications competitive. Both the representation of country-level structure and the functional form of the model therefore influenced predictive performance.

Third, simple benchmarks should be included routinely. The persistence benchmark was difficult to outperform in the primary test period, despite requiring no macroeconomic or health-system predictors. Without such a benchmark, the performance of more complex models could appear stronger than it actually is. For slowly changing population-health outcomes, recent outcome history can provide a demanding but informative reference for evaluating whether additional modeling complexity adds useful predictive information.

Finally, the limited and non-robust within-country associations for several health-system inputs caution against interpreting national expenditure or capacity indicators as direct policy effects. Previous research similarly indicates that the relationship between resources and health outcomes depends on how resources are financed, organized, and delivered (5, 10, 31). For comparative health services research, this supports combining quantitative indicators of resource levels with measures that better capture access, quality, organization, and institutional context when such data are available.

### 4.4 Limitations

Several limitations should be acknowledged. First, the analysis is based on aggregated country-level indicators, so associations observed at the country-year level should not be interpreted as relationships that necessarily hold at the individual level. Second, the study is observational. Although the fixed effects specification accounts for stable country-specific characteristics and common year effects, it does not remove bias from time-varying confounding, reverse causality, or other omitted dynamics. The one-year lagged specification was therefore used only as a sensitivity analysis of coefficient direction and not as a causal identification strategy.

Third, the dataset is small for machine-learning applications. The predictive models were trained on approximately 450 country-year observations, which limits the amount of information available to estimate more flexible relationships. The MLP was particularly sensitive to random initialization in this setting, and the ML results should therefore be interpreted as evidence from this small macro-panel rather than as an estimate of the maximum performance these algorithms could achieve with larger datasets.

Fourth, the primary 2018–2022 test period includes the COVID-19 pandemic. Although the alternative pre-pandemic analysis showed that the main conclusions were not limited to the pandemic-affected window, model rankings differed across evaluation periods. Conclusions about the relative performance of individual models should therefore be understood as specific to the temporal evaluation design used in this study.

Fifth, the study includes 25 EU countries and focuses on a single outcome, infant mortality. The findings should therefore not be generalized to other countries, health outcomes, or macro-panel settings without further replication.

Sixth, statistical inference in the fixed effects analysis is based on only 25 country clusters. Conventional cluster-robust inference may be less reliable with a limited number of clusters. The wild-cluster bootstrap sensitivity analysis showed that the two coefficients that were significant under conventional clustered inference were not statistically significant under the small-cluster procedure. The explanatory associations should therefore be interpreted cautiously.

Finally, the machine-learning analysis focused primarily on predictive performance and did not include *post hoc* interpretability methods such as SHAP values or partial dependence plots. These approaches could be considered in future work to examine how individual predictors contribute to nonlinear model predictions.

## 5 Conclusion

In this balanced panel of 25 EU countries over 2000–2022, the strong within-panel fit of the fixed effects model did not translate into strong out-of-period prediction. When the models were estimated on the same analytical sample, given comparable information where feasible, tuned using temporal cross-validation, and evaluated on the same held-out period, the tuned tree-based models performed better than the prediction-compatible fixed effects and linear specifications. Even so, the simple persistence benchmark achieved the best predictive performance in the primary 2018–2022 test period.

Adding country information improved the performance of Random Forest and Gradient Boosting, indicating that country identity contributed predictive information beyond the ten observed predictors. Country information alone, however, was not enough to make the linear specifications competitive with the nonlinear models. Model rankings also changed across evaluation periods. In the alternative pre-pandemic test period, the tuned nonlinear models performed better than persistence. This indicates that predictive performance in this panel depended not only on model type, but also on how country-level differences were represented and on the period used for evaluation.

The explanatory fixed effects analysis also requires cautious interpretation. GDP per capita and the total fertility rate were statistically significant under conventional country-clustered inference, but neither remained significant under the small-cluster wild-bootstrap sensitivity analysis. These estimates should therefore be interpreted as adjusted within-country associations rather than as evidence of causal effects.

The main conclusion is that fixed effects and machine-learning models serve different purposes and should be assessed accordingly. In this study, fixed effects were useful for examining within-country associations, while the tree-based models were more effective for out-of-period prediction. The strong performance of the persistence benchmark also shows why simple outcome-history benchmarks should be included before attributing predictive gains to more complex models. Future research could examine whether these findings hold for other health outcomes and countries, use additional temporal evaluation windows, extend the panel analysis, and explore how individual predictors contribute to nonlinear predictions.

## Data Availability

The original contributions presented in the study are included in the article/Supplementary Material, further inquiries can be directed to the corresponding author.
