# Supplementary material for: Infant mortality across EU health systems: heterogeneity encoding, fixed effects regression, and machine learning in a small macro panel
Source: Front Health Serv. 2026 Sep 16;6:1893109. doi: 10.3389/frhs.2026.1893109 (PMC13624104; doi:10.3389/frhs.2026.1893109)
Supplement: Supplementary file 1 [file Table1.docx]

**Supplementary Material**

**Supplementary Table S1. Two-way fixed effects regression coefficients (country-clustered standard errors).**

| **Variable** | **Coefficient** | **Clustered SE** | **z statistic** | **p value** | **95% CI Lower** | **95% CI Upper** |
| --- | --- | --- | --- | --- | --- | --- |
| GDP per capita | -7.31e-5 | 3.51e-5 | -2.085 | 0.037 | -0.000142 | -0.000004 |
| Health expenditure per capita | 3.80e-4 | 3.37e-4 | 1.127 | 0.260 | -0.000281 | 0.001 |
| Physicians per 1,000 | -0.2778 | 0.3037 | -0.915 | 0.360 | -0.873 | 0.317 |
| Nurses per 1,000 | 0.1631 | 0.0885 | 1.843 | 0.065 | -0.010 | 0.337 |
| Hospital beds per 1,000 | -0.0464 | 0.2427 | -0.191 | 0.848 | -0.522 | 0.429 |
| Total fertility rate | -3.4972 | 1.6058 | -2.178 | 0.029 | -6.645 | -0.350 |
| Female unemployment | -0.0206 | 0.0380 | -0.543 | 0.587 | -0.095 | 0.054 |
| Alcohol consumption | -0.1393 | 0.3482 | -0.400 | 0.689 | -0.822 | 0.543 |
| DTP vaccination coverage | 0.1327 | 0.0776 | 1.710 | 0.087 | -0.019 | 0.285 |
| Female tertiary education | -0.0011 | 0.0103 | -0.110 | 0.912 | -0.021 | 0.019 |

*Note.* Coefficients are adjusted within-country associations after absorbing country and year fixed effects; standard errors are clustered by country. Country and year indicator coefficients are omitted for readability.

**Supplementary Table S1 (continued). Fixed effects model summary statistics.**

| **Statistic** | **Value** |
| --- | --- |
| Dependent variable | Infant mortality rate |
| Number of observations | 575 |
| Full-model R² | 0.882 |
| Adjusted R² | 0.869 |
| Fixed-effects-only R² (no covariates) | 0.814 |
| Partial R² of covariate block net of FE | 0.366 |
| Joint F-test of 10 covariates (net of FE) | 3.212 |
| Joint F-test p value | 0.009 |
| Pooled OLS R² (same sample) | 0.531 |

*Note.* The full-model R² includes the country and year indicators. The partial R² represents the incremental explanatory contribution of the ten substantive covariates conditional on the country and year fixed effects. The joint F-test uses country-clustered inference.

**Supplementary Table S1 (continued). Nested indicator specifications on the identical 575-observation sample.**

| **Specification** | **R²** | **Adjusted R²** |
| --- | --- | --- |
| Pooled OLS (no indicators) | 0.531 | 0.523 |
| OLS + country indicators | 0.863 | 0.854 |
| OLS + year indicators | 0.552 | 0.526 |
| OLS + country & year (two-way FE/LSDV) | 0.882 | 0.869 |

*Note.* All four models include the ten substantive covariates and differ only in the indicator sets included. Adding country indicators increased R² from 0.531 to 0.863, whereas adding year indicators alone increased R² from 0.531 to 0.552. The specification containing both country and year indicators produced R² = 0.882.

**Supplementary Table S1 (continued). Small-cluster inference for the two covariates significant under conventional country-clustered inference.**

| **Variable** | **Coefficient** | **Cluster-robust p** | **Wild-cluster bootstrap p** |
| --- | --- | --- | --- |
| gdp per capita | -7.31e-5 | 0.037 | 0.156 |
| fertility rate | -3.4972 | 0.029 | 0.095 |

*Note.* Because the panel contains 25 country clusters, inference for the two covariates significant under conventional country-clustered inference was re-examined using a wild-cluster bootstrap with Rademacher weights, a restricted null, and 9,999 replications. The two-sided bootstrap p values were 0.156 for GDP per capita and 0.095 for the total fertility rate. Neither coefficient therefore remained statistically significant at the 5% level under the small-cluster sensitivity analysis.

**Supplementary Table S2. One-year lagged-predictor fixed effects sensitivity analysis.**

| **Variable** | **Main coef.** | **Main p** | **Lagged coef.** | **Lagged p** | **Same sign** |
| --- | --- | --- | --- | --- | --- |
| GDP per capita | -7.31e-5 | 0.037 | -7.39e-5 | 0.036 | Yes |
| Health expenditure per capita | 3.80e-4 | 0.260 | 3.36e-4 | 0.307 | Yes |
| Physicians per 1,000 | -0.2778 | 0.360 | -0.2034 | 0.476 | Yes |
| Nurses per 1,000 | 0.1631 | 0.065 | 0.1721 | 0.058 | Yes |
| Hospital beds per 1,000 | -0.0464 | 0.848 | -0.0666 | 0.773 | Yes |
| Total fertility rate | -3.4972 | 0.029 | -3.2719 | 0.031 | Yes |
| Female unemployment | -0.0206 | 0.587 | -0.0204 | 0.589 | Yes |
| Alcohol consumption | -0.1393 | 0.689 | -0.1340 | 0.716 | Yes |
| DTP vaccination coverage | 0.1327 | 0.087 | 0.1265 | 0.094 | Yes |
| Female tertiary education | -0.0011 | 0.912 | -0.0027 | 0.800 | Yes |

*Note.* The lagged specification is a sign-stability sensitivity analysis and is not interpreted as resolving endogeneity or establishing causal identification. All ten coefficients retained their sign under one-year lagging.

**Supplementary Table S3. Included countries and coverage (25 EU countries, 2000–2022).**

| **Country** | **First year** | **Last year** | **No. of Years** | **Country** | **First year** | **Last year** | **No. of Years** |
| --- | --- | --- | --- | --- | --- | --- | --- |
| Austria | 2000 | 2022 | 23 | Italy | 2000 | 2022 | 23 |
| Belgium | 2000 | 2022 | 23 | Latvia | 2000 | 2022 | 23 |
| Bulgaria | 2000 | 2022 | 23 | Lithuania | 2000 | 2022 | 23 |
| Croatia | 2000 | 2022 | 23 | Luxembourg | 2000 | 2022 | 23 |
| Czechia | 2000 | 2022 | 23 | Netherlands | 2000 | 2022 | 23 |
| Denmark | 2000 | 2022 | 23 | Poland | 2000 | 2022 | 23 |
| Estonia | 2000 | 2022 | 23 | Portugal | 2000 | 2022 | 23 |
| Finland | 2000 | 2022 | 23 | Romania | 2000 | 2022 | 23 |
| France | 2000 | 2022 | 23 | Slovak Republic | 2000 | 2022 | 23 |
| Germany | 2000 | 2022 | 23 | Slovenia | 2000 | 2022 | 23 |
| Greece | 2000 | 2022 | 23 | Spain | 2000 | 2022 | 23 |
| Hungary | 2000 | 2022 | 23 | Sweden | 2000 | 2022 | 23 |
| Ireland | 2000 | 2022 | 23 |  |  |  |  |

*Note.* Cyprus and Malta, the two remaining EU-27 member states, were excluded before analysis because of insufficient data coverage across several required variables. The resulting panel of 25 countries is balanced and complete, with 23 annual observations per country from 2000 to 2022 (575 country-year observations).

**Supplementary Table S4. Sample construction and missingness.**

| **Step** | **Countries** | **Years** | **Observations** | **Missing values** |
| --- | --- | --- | --- | --- |
| EU member states | 27 | — | — | — |
| Exclude Cyprus and Malta (insufficient coverage) | 25 | 23 | 575 | 0 |
| Final analytic sample (all models) | 25 | 23 | 575 | 0 |

*Note.* Cyprus and Malta were excluded during dataset construction because complete longitudinal series across the required variables could not be assembled for them over 2000–2022. For the 25 included countries, the analytic panel is fully balanced (25 × 23 = 575) with no missing values in the included variables. No imputation was therefore required, and all analyses were based on the same 575-observation panel. For the predictive analysis, the training period was 2000–2017 (450 observations) and the test period was 2018–2022 (125 observations).

**Supplementary Table S5. Variance inflation factors: pooled and two-way within.**

| **Variable** | **Pooled VIF** | **Within VIF** |
| --- | --- | --- |
| Health expenditure per capita | 4.36 | 1.27 |
| GDP per capita | 4.33 | 1.39 |
| Nurses per 1,000 | 2.78 | 1.20 |
| Alcohol consumption | 1.82 | 1.21 |
| Female unemployment | 1.79 | 1.49 |
| Female tertiary education | 1.75 | 1.17 |
| Hospital beds per 1,000 | 1.63 | 1.42 |
| Physicians per 1,000 | 1.60 | 1.24 |
| Total fertility rate | 1.58 | 1.61 |
| DTP vaccination coverage | 1.22 | 1.14 |

*Note.* Pooled VIFs were computed on the untransformed predictors, while within VIFs were computed after the two-way country-and-year within transformation used by the fixed effects estimator. VIF was used as a diagnostic for the regression-based specifications and not as a model-validity criterion for the tree-based learners. Values of 5 and 10 are commonly used as rule-of-thumb thresholds for potentially problematic multicollinearity, although such cutoffs should not be interpreted rigidly (O’Brien, 2007).

**Supplementary Table S6. Hyperparameter search spaces, selected configurations, and cross-validation structure.**

Hyperparameters were selected using expanding-window (rolling-origin) temporal cross-validation within the 2000–2017 training period. Validation was conducted using year-based folds. For each validation year in {2013, 2014, 2015, 2016, 2017}, all years preceding the validation year formed the training fold, and the entire validation year, including all countries, was held out. Models were selected based on root mean squared error (RMSE), and the 2018–2022 test period remained untouched until final evaluation. For the alternative pre-pandemic split (Table 5), the same procedure was applied using validation years {2010, 2011, 2012, 2013, 2014}. The search spaces, selected configurations, and cross-validated RMSE values are reported below.

| **Model** | **Search space (tuned parameters)** | **Selected configuration** | **CV RMSE** |
| --- | --- | --- | --- |
| Random Forest (no country) | n_estimators {300, 500}; max_depth {None, 6, 10}; min_samples_leaf {1, 2, 4}; max_features {sqrt, 0.7} | n_estimators=500, max_depth=None, min_samples_leaf=1, max_features=sqrt | 0.509 |
| Random Forest (+ country) | n_estimators {300, 500}; max_depth {None, 6, 10}; min_samples_leaf {1, 2, 4}; max_features {sqrt, 0.7} | n_estimators=300, max_depth=None, min_samples_leaf=1, max_features=sqrt | 0.496 |
| Gradient Boosting (no country) | n_estimators {100, 300}; learning_rate {0.03, 0.05, 0.10}; max_depth {2, 3}; subsample {0.8, 1.0} | n_estimators=300, learning_rate=0.1, max_depth=3, subsample=0.8 | 0.533 |
| Gradient Boosting (+ country) | n_estimators {100, 300}; learning_rate {0.03, 0.05, 0.10}; max_depth {2, 3}; subsample {0.8, 1.0} | n_estimators=300, learning_rate=0.1, max_depth=3, subsample=1.0 | 0.454 |
| MLP (no country) | hidden_layers {(32,), (64, 32)}; alpha {1e-4, 1e-3, 1e-2}; learning_rate_init {5e-4, 1e-3} | hidden_layers=(64, 32), alpha=0.01, learning_rate_init=0.0005 | 0.525 |
| MLP (+ country) | hidden_layers {(32,), (64, 32)}; alpha {1e-4, 1e-3, 1e-2}; learning_rate_init {5e-4, 1e-3} | hidden_layers=(32,), alpha=0.001, learning_rate_init=0.001 | 0.443 |

*Note.* Selected configurations correspond to the lowest cross-validated RMSE within the 2000–2017 training period. Because the MLP is stochastic, the tuned country-informed MLP was refitted across ten random seeds (Supplementary Table S8). The value reported in Table 2 corresponds to seed 42.

**Supplementary Table S7a. Predictive performance by period within the primary 2018–2022 window.**

| **Period** | **Model** | **RMSE** | **MAE** | **R²** |
| --- | --- | --- | --- | --- |
| **Full test 2018–2022** | Persistence benchmark | 0.534 | 0.382 | 0.737 |
|  | Historical country-mean benchmark | 2.094 | 1.511 | −3.055 |
|  | Linear regression (no country) | 1.835 | 1.499 | −2.114 |
|  | Random Forest (no country) | 0.631 | 0.504 | 0.632 |
|  | Random Forest (+ country) | 0.578 | 0.470 | 0.691 |
|  | Gradient Boosting (no country) | 0.700 | 0.536 | 0.547 |
|  | Gradient Boosting (+ country) | 0.559 | 0.428 | 0.711 |
|  | MLP (no country) | 0.679 | 0.530 | 0.574 |
|  | MLP (+ country) | 0.761 | 0.645 | 0.464 |
|  | Two-way FE, last-year-effect rule | 1.333 | 0.979 | −0.645 |
|  | Country FE + linear trend | 1.442 | 1.120 | −0.924 |
|  | Cobb-Douglas | 1.067 | 0.841 | −0.053 |
| **Pre-pandemic 2018–2019** | Persistence benchmark | 0.477 | 0.336 | 0.806 |
|  | Historical country-mean benchmark | 1.995 | 1.425 | −2.392 |
|  | Linear regression (no country) | 1.643 | 1.369 | −1.301 |
|  | Random Forest (no country) | 0.605 | 0.482 | 0.688 |
|  | Random Forest (+ country) | 0.553 | 0.429 | 0.739 |
|  | Gradient Boosting (no country) | 0.688 | 0.545 | 0.596 |
|  | Gradient Boosting (+ country) | 0.557 | 0.423 | 0.736 |
|  | MLP (no country) | 0.535 | 0.419 | 0.756 |
|  | MLP (+ country) | 0.558 | 0.467 | 0.735 |
|  | Two-way FE, last-year-effect rule | 1.115 | 0.858 | −0.060 |
|  | Country FE + linear trend | 1.245 | 1.036 | −0.321 |
|  | Cobb-Douglas | 1.130 | 0.891 | −0.089 |
| **Pandemic-affected 2020–2022** | Persistence benchmark | 0.569 | 0.413 | 0.678 |
|  | Historical country-mean benchmark | 2.157 | 1.568 | −3.631 |
|  | Linear regression (no country) | 1.952 | 1.586 | −2.793 |
|  | Random Forest (no country) | 0.647 | 0.518 | 0.584 |
|  | Random Forest (+ country) | 0.593 | 0.497 | 0.650 |
|  | Gradient Boosting (no country) | 0.707 | 0.530 | 0.502 |
|  | Gradient Boosting (+ country) | 0.561 | 0.432 | 0.687 |
|  | MLP (no country) | 0.760 | 0.605 | 0.425 |
|  | MLP (+ country) | 0.871 | 0.763 | 0.245 |
|  | Two-way FE, last-year-effect rule | 1.461 | 1.059 | −1.124 |
|  | Country FE + linear trend | 1.560 | 1.176 | −1.422 |
|  | Cobb-Douglas | 1.022 | 0.807 | −0.040 |

*Note.* All models were trained on 2000–2017. The 2018–2022 test predictions were then summarized for the full test period, the pre-pandemic period (2018–2019), and the pandemic-affected period (2020–2022). No model was re-estimated for these within-test-period comparisons.

**Supplementary Table S7b. Predictive performance by test year (primary split).**

| **Year** | **Model** | **RMSE** | **MAE** | **R²** |
| --- | --- | --- | --- | --- |
| **2018** | Persistence benchmark | 0.433 | 0.324 | 0.840 |
|  | Historical country-mean benchmark | 1.972 | 1.393 | −2.324 |
|  | Linear regression (no country) | 1.613 | 1.371 | −1.224 |
|  | Random Forest (no country) | 0.574 | 0.460 | 0.718 |
|  | Random Forest (+ country) | 0.521 | 0.410 | 0.768 |
|  | Gradient Boosting (no country) | 0.658 | 0.492 | 0.630 |
|  | Gradient Boosting (+ country) | 0.534 | 0.396 | 0.756 |
|  | MLP (no country) | 0.509 | 0.413 | 0.779 |
|  | MLP (+ country) | 0.458 | 0.415 | 0.821 |
|  | Two-way FE, last-year-effect rule | 1.045 | 0.802 | 0.068 |
|  | Country FE + linear trend | 1.175 | 0.952 | −0.180 |
|  | Cobb–Douglas | 1.175 | 0.936 | −0.180 |
| **2019** | Persistence benchmark | 0.517 | 0.348 | 0.773 |
|  | Historical country-mean benchmark | 2.017 | 1.457 | −2.460 |
|  | Linear regression (no country) | 1.672 | 1.366 | −1.378 |
|  | Random Forest (no country) | 0.635 | 0.504 | 0.657 |
|  | Random Forest (+ country) | 0.583 | 0.448 | 0.711 |
|  | Gradient Boosting (no country) | 0.717 | 0.599 | 0.563 |
|  | Gradient Boosting (+ country) | 0.579 | 0.450 | 0.715 |
|  | MLP (no country) | 0.559 | 0.425 | 0.734 |
|  | MLP (+ country) | 0.643 | 0.520 | 0.648 |
|  | Two-way FE, last-year-effect rule | 1.181 | 0.914 | −0.187 |
|  | Country FE + linear trend | 1.311 | 1.119 | −0.462 |
|  | Cobb–Douglas | 1.084 | 0.847 | 0.001 |
| **2020** | Persistence benchmark | 0.541 | 0.396 | 0.719 |
|  | Historical country-mean benchmark | 2.127 | 1.553 | −3.333 |
|  | Linear regression (no country) | 1.837 | 1.503 | −2.234 |
|  | Random Forest (no country) | 0.610 | 0.481 | 0.643 |
|  | Random Forest (+ country) | 0.572 | 0.474 | 0.687 |
|  | Gradient Boosting (no country) | 0.717 | 0.513 | 0.507 |
|  | Gradient Boosting (+ country) | 0.583 | 0.438 | 0.674 |
|  | MLP (no country) | 0.665 | 0.509 | 0.577 |
|  | MLP (+ country) | 0.779 | 0.667 | 0.419 |
|  | Two-way FE, last-year-effect rule | 1.256 | 1.001 | −0.512 |
|  | Country FE + linear trend | 1.199 | 0.992 | −0.376 |
|  | Cobb–Douglas | 1.137 | 0.905 | −0.238 |
| **2021** | Persistence benchmark | 0.532 | 0.400 | 0.713 |
|  | Historical country-mean benchmark | 2.176 | 1.612 | −3.789 |
|  | Linear regression (no country) | 1.952 | 1.586 | −2.857 |
|  | Random Forest (no country) | 0.610 | 0.504 | 0.623 |
|  | Random Forest (+ country) | 0.545 | 0.468 | 0.700 |
|  | Gradient Boosting (no country) | 0.604 | 0.489 | 0.631 |
|  | Gradient Boosting (+ country) | 0.482 | 0.405 | 0.765 |
|  | MLP (no country) | 0.698 | 0.605 | 0.507 |
|  | MLP (+ country) | 0.886 | 0.767 | 0.206 |
|  | Two-way FE, last-year-effect rule | 1.536 | 1.068 | −1.387 |
|  | Country FE + linear trend | 1.674 | 1.183 | −1.835 |
|  | Cobb–Douglas | 0.974 | 0.778 | 0.041 |
| **2022** | Persistence benchmark | 0.627 | 0.444 | 0.596 |
|  | Historical country-mean benchmark | 2.169 | 1.540 | −3.837 |
|  | Linear regression (no country) | 2.061 | 1.670 | −3.366 |
|  | Random Forest (no country) | 0.715 | 0.570 | 0.475 |
|  | Random Forest (+ country) | 0.658 | 0.548 | 0.555 |
|  | Gradient Boosting (no country) | 0.788 | 0.588 | 0.361 |
|  | Gradient Boosting (+ country) | 0.610 | 0.454 | 0.618 |
|  | MLP (no country) | 0.897 | 0.701 | 0.174 |
|  | MLP (+ country) | 0.940 | 0.856 | 0.091 |
|  | Two-way FE, last-year-effect rule | 1.571 | 1.109 | −1.536 |
|  | Country FE + linear trend | 1.751 | 1.353 | −2.150 |
|  | Cobb–Douglas | 0.946 | 0.739 | 0.080 |

*Note.* Metrics for each year are based on the 25 country observations in that test year. All models were trained on 2000–2017 and were not re-estimated separately for individual test years. RMSE = root mean squared error; MAE = mean absolute error; R² = coefficient of determination.

**Supplementary Table S8. Country-informed MLP test performance across ten random seeds.**

| **Seed** | **Test RMSE** | **Test MAE** | **Test R²** |
| --- | --- | --- | --- |
| 1 | 0.762 | 0.615 | 0.462 |
| 7 | 0.662 | 0.539 | 0.595 |
| 21 | 0.744 | 0.616 | 0.489 |
| 42 | 0.761 | 0.645 | 0.464 |
| 84 | 0.789 | 0.645 | 0.425 |
| 123 | 0.809 | 0.657 | 0.395 |
| 202 | 0.749 | 0.589 | 0.481 |
| 555 | 0.914 | 0.744 | 0.227 |
| 777 | 0.690 | 0.540 | 0.559 |
| 2026 | 0.677 | 0.525 | 0.576 |

**Supplementary Table S8 (continued). Summary across seeds.**

| **Metric** | **Mean** | **SD** | **Minimum** | **Maximum** |
| --- | --- | --- | --- | --- |
| RMSE | 0.756 | 0.073 | 0.662 | 0.914 |
| MAE | 0.611 | 0.067 | 0.525 | 0.744 |
| R2 | 0.467 | 0.106 | 0.227 | 0.595 |

*Note.* The tuned country-informed MLP, with architecture selected by temporal cross-validation (Supplementary Table S6), was refitted using ten random seeds and evaluated on the unchanged 2018–2022 test period. The seed-42 result is the value reported in Table 2. RMSE = root mean squared error; MAE = mean absolute error; R² = coefficient of determination..

**Supplementary Table S9. Observed and predicted infant mortality rates with signed and absolute errors, all test-period country-years.**

For each test-period country-year, the table reports the observed infant mortality rate and, for each of the four principal models, the prediction, the signed error (predicted minus observed), and the absolute error. Persistence = country-specific 2017 value carried forward; RF+C and GB+C = tuned Random Forest and Gradient Boosting with country information; FE(LY) = two-way fixed effects under the last-year-effect rule. Predictions and errors are in deaths per 1,000 live births. Predictions and both error columns for all predictive models, fixed-effects specifications, and benchmarks (including the predictor-only learners, the MLP specifications, the prediction-compatible fixed-effects specifications, and the linear and Cobb-Douglas benchmarks) are provided in Supplementary Data File S1.

| **Country** | **Yr** | **Obs** | **Pers** | **s** | **\|e\|** | **RF+C** | **s** | **\|e\|** | **GB+C** | **s** | **\|e\|** | **FE(LY)** | **s** | **\|e\|** |
| --- | --- | --- | --- | --- | --- | --- | --- | --- | --- | --- | --- | --- | --- | --- |
| Austria | 2018 | 2.70 | 2.90 | +0.20 | 0.20 | 3.14 | +0.44 | 0.44 | 2.98 | +0.28 | 0.28 | 2.36 | -0.34 | 0.34 |
| Austria | 2019 | 2.90 | 2.90 | +0.00 | 0.00 | 3.27 | +0.37 | 0.37 | 3.25 | +0.35 | 0.35 | 2.77 | -0.13 | 0.13 |
| Austria | 2020 | 3.10 | 2.90 | -0.20 | 0.20 | 3.23 | +0.13 | 0.13 | 2.93 | -0.17 | 0.17 | 3.44 | +0.34 | 0.34 |
| Austria | 2021 | 2.70 | 2.90 | +0.20 | 0.20 | 3.19 | +0.49 | 0.49 | 2.87 | +0.17 | 0.17 | 3.51 | +0.81 | 0.81 |
| Austria | 2022 | 2.40 | 2.90 | +0.50 | 0.50 | 3.25 | +0.85 | 0.85 | 2.78 | +0.38 | 0.38 | 2.82 | +0.42 | 0.42 |
| Belgium | 2018 | 3.80 | 3.60 | -0.20 | 0.20 | 3.38 | -0.42 | 0.42 | 3.46 | -0.34 | 0.34 | 4.04 | +0.24 | 0.24 |
| Belgium | 2019 | 3.70 | 3.60 | -0.10 | 0.10 | 3.39 | -0.31 | 0.31 | 3.14 | -0.56 | 0.56 | 3.88 | +0.18 | 0.18 |
| Belgium | 2020 | 3.30 | 3.60 | +0.30 | 0.30 | 3.33 | +0.03 | 0.03 | 2.85 | -0.45 | 0.45 | 4.54 | +1.24 | 1.24 |
| Belgium | 2021 | 2.90 | 3.60 | +0.70 | 0.70 | 3.24 | +0.34 | 0.34 | 3.12 | +0.22 | 0.22 | 4.10 | +1.20 | 1.20 |
| Belgium | 2022 | 2.90 | 3.60 | +0.70 | 0.70 | 3.24 | +0.34 | 0.34 | 2.88 | -0.02 | 0.02 | 3.90 | +1.00 | 1.00 |
| Bulgaria | 2018 | 5.80 | 6.40 | +0.60 | 0.60 | 6.26 | +0.46 | 0.46 | 7.10 | +1.30 | 1.30 | 7.25 | +1.45 | 1.45 |
| Bulgaria | 2019 | 5.60 | 6.40 | +0.80 | 0.80 | 5.57 | -0.03 | 0.03 | 6.56 | +0.96 | 0.96 | 7.06 | +1.46 | 1.46 |
| Bulgaria | 2020 | 5.10 | 6.40 | +1.30 | 1.30 | 5.51 | +0.41 | 0.41 | 6.22 | +1.12 | 1.12 | 7.21 | +2.11 | 2.11 |
| Bulgaria | 2021 | 5.60 | 6.40 | +0.80 | 0.80 | 5.26 | -0.34 | 0.34 | 6.52 | +0.92 | 0.92 | 6.80 | +1.20 | 1.20 |
| Bulgaria | 2022 | 4.80 | 6.40 | +1.60 | 1.60 | 5.03 | +0.23 | 0.23 | 5.36 | +0.56 | 0.56 | 6.82 | +2.02 | 2.02 |
| Croatia | 2018 | 4.20 | 4.00 | -0.20 | 0.20 | 4.02 | -0.18 | 0.18 | 3.37 | -0.83 | 0.83 | 3.71 | -0.49 | 0.49 |
| Croatia | 2019 | 4.00 | 4.00 | +0.00 | 0.00 | 4.09 | +0.09 | 0.09 | 3.51 | -0.49 | 0.49 | 3.70 | -0.30 | 0.30 |
| Croatia | 2020 | 4.00 | 4.00 | +0.00 | 0.00 | 4.11 | +0.11 | 0.11 | 3.98 | -0.02 | 0.02 | 3.91 | -0.09 | 0.09 |
| Croatia | 2021 | 3.80 | 4.00 | +0.20 | 0.20 | 3.62 | -0.18 | 0.18 | 3.15 | -0.65 | 0.65 | 3.22 | -0.58 | 0.58 |
| Croatia | 2022 | 4.10 | 4.00 | -0.10 | 0.10 | 3.21 | -0.89 | 0.89 | 2.99 | -1.11 | 1.11 | 2.96 | -1.14 | 1.14 |
| Czechia | 2018 | 2.60 | 2.70 | +0.10 | 0.10 | 2.99 | +0.39 | 0.39 | 2.78 | +0.18 | 0.18 | 0.75 | -1.85 | 1.85 |
| Czechia | 2019 | 2.60 | 2.70 | +0.10 | 0.10 | 3.05 | +0.45 | 0.45 | 3.15 | +0.55 | 0.55 | 0.61 | -1.99 | 1.99 |
| Czechia | 2020 | 2.30 | 2.70 | +0.40 | 0.40 | 3.05 | +0.75 | 0.75 | 2.95 | +0.65 | 0.65 | 1.09 | -1.21 | 1.21 |
| Czechia | 2021 | 2.20 | 2.70 | +0.50 | 0.50 | 3.20 | +1.00 | 1.00 | 3.00 | +0.80 | 0.80 | 0.37 | -1.83 | 1.83 |
| Czechia | 2022 | 2.30 | 2.70 | +0.40 | 0.40 | 3.11 | +0.81 | 0.81 | 2.96 | +0.66 | 0.66 | 0.74 | -1.56 | 1.56 |
| Denmark | 2018 | 3.10 | 3.20 | +0.10 | 0.10 | 3.20 | +0.10 | 0.10 | 3.29 | +0.19 | 0.19 | 3.52 | +0.42 | 0.42 |
| Denmark | 2019 | 2.10 | 3.20 | +1.10 | 1.10 | 3.20 | +1.10 | 1.10 | 3.34 | +1.24 | 1.24 | 3.55 | +1.45 | 1.45 |
| Denmark | 2020 | 2.40 | 3.20 | +0.80 | 0.80 | 3.17 | +0.77 | 0.77 | 3.27 | +0.87 | 0.87 | 3.88 | +1.48 | 1.48 |
| Denmark | 2021 | 2.40 | 3.20 | +0.80 | 0.80 | 3.17 | +0.77 | 0.77 | 3.12 | +0.72 | 0.72 | 3.41 | +1.01 | 1.01 |
| Denmark | 2022 | 2.30 | 3.20 | +0.90 | 0.90 | 3.15 | +0.85 | 0.85 | 2.97 | +0.67 | 0.67 | 3.66 | +1.36 | 1.36 |
| Estonia | 2018 | 1.60 | 2.30 | +0.70 | 0.70 | 2.94 | +1.34 | 1.34 | 2.73 | +1.13 | 1.13 | 2.45 | +0.85 | 0.85 |
| Estonia | 2019 | 1.60 | 2.30 | +0.70 | 0.70 | 2.90 | +1.30 | 1.30 | 2.67 | +1.07 | 1.07 | 2.32 | +0.72 | 0.72 |
| Estonia | 2020 | 1.40 | 2.30 | +0.90 | 0.90 | 2.67 | +1.27 | 1.27 | 2.29 | +0.89 | 0.89 | 2.88 | +1.48 | 1.48 |
| Estonia | 2021 | 2.20 | 2.30 | +0.10 | 0.10 | 2.80 | +0.60 | 0.60 | 2.33 | +0.13 | 0.13 | 2.37 | +0.17 | 0.17 |
| Estonia | 2022 | 2.20 | 2.30 | +0.10 | 0.10 | 3.03 | +0.83 | 0.83 | 2.63 | +0.43 | 0.43 | 2.36 | +0.16 | 0.16 |
| Finland | 2018 | 2.10 | 2.00 | -0.10 | 0.10 | 2.30 | +0.20 | 0.20 | 2.04 | -0.06 | 0.06 | 2.53 | +0.43 | 0.43 |
| Finland | 2019 | 2.10 | 2.00 | -0.10 | 0.10 | 2.46 | +0.36 | 0.36 | 2.25 | +0.15 | 0.15 | 2.79 | +0.69 | 0.69 |
| Finland | 2020 | 1.80 | 2.00 | +0.20 | 0.20 | 2.40 | +0.60 | 0.60 | 2.00 | +0.20 | 0.20 | 2.77 | +0.97 | 0.97 |
| Finland | 2021 | 1.80 | 2.00 | +0.20 | 0.20 | 2.49 | +0.69 | 0.69 | 2.13 | +0.33 | 0.33 | 2.46 | +0.66 | 0.66 |
| Finland | 2022 | 2.00 | 2.00 | +0.00 | 0.00 | 2.63 | +0.63 | 0.63 | 2.09 | +0.09 | 0.09 | 3.24 | +1.24 | 1.24 |
| France | 2018 | 3.80 | 3.90 | +0.10 | 0.10 | 3.75 | -0.05 | 0.05 | 3.73 | -0.07 | 0.07 | 3.59 | -0.21 | 0.21 |
| France | 2019 | 3.80 | 3.90 | +0.10 | 0.10 | 3.65 | -0.15 | 0.15 | 3.68 | -0.12 | 0.12 | 3.54 | -0.26 | 0.26 |
| France | 2020 | 3.60 | 3.90 | +0.30 | 0.30 | 3.38 | -0.22 | 0.22 | 3.38 | -0.22 | 0.22 | 3.91 | +0.31 | 0.31 |
| France | 2021 | 3.70 | 3.90 | +0.20 | 0.20 | 3.35 | -0.35 | 0.35 | 3.12 | -0.58 | 0.58 | 3.75 | +0.05 | 0.05 |
| France | 2022 | 4.00 | 3.90 | -0.10 | 0.10 | 3.25 | -0.75 | 0.75 | 3.18 | -0.82 | 0.82 | 3.64 | -0.36 | 0.36 |
| Germany | 2018 | 3.20 | 3.30 | +0.10 | 0.10 | 3.42 | +0.22 | 0.22 | 3.34 | +0.14 | 0.14 | 1.95 | -1.25 | 1.25 |
| Germany | 2019 | 3.20 | 3.30 | +0.10 | 0.10 | 3.45 | +0.25 | 0.25 | 3.43 | +0.23 | 0.23 | 2.07 | -1.13 | 1.13 |
| Germany | 2020 | 3.10 | 3.30 | +0.20 | 0.20 | 3.43 | +0.33 | 0.33 | 3.44 | +0.34 | 0.34 | 2.66 | -0.44 | 0.44 |
| Germany | 2021 | 3.00 | 3.30 | +0.30 | 0.30 | 3.36 | +0.36 | 0.36 | 3.26 | +0.26 | 0.26 | 2.55 | -0.45 | 0.45 |
| Germany | 2022 | 3.20 | 3.30 | +0.10 | 0.10 | 3.40 | +0.20 | 0.20 | 3.26 | +0.06 | 0.06 | 2.44 | -0.76 | 0.76 |
| Greece | 2018 | 3.50 | 3.50 | +0.00 | 0.00 | 3.71 | +0.21 | 0.21 | 3.51 | +0.01 | 0.01 | 3.31 | -0.19 | 0.19 |
| Greece | 2019 | 3.70 | 3.50 | -0.20 | 0.20 | 3.70 | +0.00 | 0.00 | 3.50 | -0.20 | 0.20 | 3.28 | -0.42 | 0.42 |
| Greece | 2020 | 3.20 | 3.50 | +0.30 | 0.30 | 3.75 | +0.55 | 0.55 | 3.91 | +0.71 | 0.71 | 3.59 | +0.39 | 0.39 |
| Greece | 2021 | 3.50 | 3.50 | +0.00 | 0.00 | 3.62 | +0.12 | 0.12 | 3.54 | +0.04 | 0.04 | 3.14 | -0.36 | 0.36 |
| Greece | 2022 | 3.00 | 3.50 | +0.50 | 0.50 | 3.59 | +0.59 | 0.59 | 2.87 | -0.13 | 0.13 | 2.93 | -0.07 | 0.07 |
| Hungary | 2018 | 3.30 | 3.50 | +0.20 | 0.20 | 3.93 | +0.63 | 0.63 | 3.77 | +0.47 | 0.47 | 3.62 | +0.32 | 0.32 |
| Hungary | 2019 | 3.60 | 3.50 | -0.10 | 0.10 | 3.86 | +0.26 | 0.26 | 3.53 | -0.07 | 0.07 | 3.27 | -0.33 | 0.33 |
| Hungary | 2020 | 3.40 | 3.50 | +0.10 | 0.10 | 3.85 | +0.45 | 0.45 | 3.53 | +0.13 | 0.13 | 3.71 | +0.31 | 0.31 |
| Hungary | 2021 | 3.30 | 3.50 | +0.20 | 0.20 | 3.78 | +0.48 | 0.48 | 3.46 | +0.16 | 0.16 | 3.33 | +0.03 | 0.03 |
| Hungary | 2022 | 3.60 | 3.50 | -0.10 | 0.10 | 3.77 | +0.17 | 0.17 | 3.57 | -0.03 | 0.03 | 3.14 | -0.46 | 0.46 |
| Ireland | 2018 | 2.90 | 3.00 | +0.10 | 0.10 | 3.22 | +0.32 | 0.32 | 3.25 | +0.35 | 0.35 | 1.29 | -1.61 | 1.61 |
| Ireland | 2019 | 2.80 | 3.00 | +0.20 | 0.20 | 3.38 | +0.58 | 0.58 | 3.17 | +0.37 | 0.37 | 1.36 | -1.44 | 1.44 |
| Ireland | 2020 | 3.10 | 3.00 | -0.10 | 0.10 | 3.31 | +0.21 | 0.21 | 2.93 | -0.17 | 0.17 | 1.55 | -1.55 | 1.55 |
| Ireland | 2021 | 3.30 | 3.00 | -0.30 | 0.30 | 3.19 | -0.11 | 0.11 | 3.17 | -0.13 | 0.13 | -1.69 | -4.99 | 4.99 |
| Ireland | 2022 | 3.50 | 3.00 | -0.50 | 0.50 | 3.35 | -0.15 | 0.15 | 2.82 | -0.68 | 0.68 | -1.49 | -4.99 | 4.99 |
| Italy | 2018 | 2.80 | 2.70 | -0.10 | 0.10 | 2.88 | +0.08 | 0.08 | 2.79 | -0.01 | 0.01 | 3.20 | +0.40 | 0.40 |
| Italy | 2019 | 2.40 | 2.70 | +0.30 | 0.30 | 2.97 | +0.57 | 0.57 | 2.77 | +0.37 | 0.37 | 3.44 | +1.04 | 1.04 |
| Italy | 2020 | 2.40 | 2.70 | +0.30 | 0.30 | 2.99 | +0.59 | 0.59 | 2.68 | +0.28 | 0.28 | 3.84 | +1.44 | 1.44 |
| Italy | 2021 | 2.30 | 2.70 | +0.40 | 0.40 | 3.03 | +0.73 | 0.73 | 2.76 | +0.46 | 0.46 | 2.77 | +0.47 | 0.47 |
| Italy | 2022 | 2.30 | 2.70 | +0.40 | 0.40 | 3.02 | +0.72 | 0.72 | 2.44 | +0.14 | 0.14 | 2.52 | +0.22 | 0.22 |
| Latvia | 2018 | 3.20 | 4.10 | +0.90 | 0.90 | 4.01 | +0.81 | 0.81 | 3.89 | +0.69 | 0.69 | 5.18 | +1.98 | 1.98 |
| Latvia | 2019 | 3.40 | 4.10 | +0.70 | 0.70 | 4.04 | +0.64 | 0.64 | 3.78 | +0.38 | 0.38 | 5.74 | +2.34 | 2.34 |
| Latvia | 2020 | 3.50 | 4.10 | +0.60 | 0.60 | 3.82 | +0.32 | 0.32 | 3.72 | +0.22 | 0.22 | 6.09 | +2.59 | 2.59 |
| Latvia | 2021 | 2.70 | 4.10 | +1.40 | 1.40 | 3.29 | +0.59 | 0.59 | 2.97 | +0.27 | 0.27 | 5.53 | +2.83 | 2.83 |
| Latvia | 2022 | 2.40 | 4.10 | +1.70 | 1.70 | 3.41 | +1.01 | 1.01 | 3.38 | +0.98 | 0.98 | 5.55 | +3.15 | 3.15 |
| Lithuania | 2018 | 3.60 | 3.00 | -0.60 | 0.60 | 3.25 | -0.35 | 0.35 | 3.26 | -0.34 | 0.34 | 3.44 | -0.16 | 0.16 |
| Lithuania | 2019 | 3.60 | 3.00 | -0.60 | 0.60 | 3.22 | -0.38 | 0.38 | 3.18 | -0.42 | 0.42 | 3.65 | +0.05 | 0.05 |
| Lithuania | 2020 | 3.00 | 3.00 | +0.00 | 0.00 | 3.34 | +0.34 | 0.34 | 3.09 | +0.09 | 0.09 | 3.97 | +0.97 | 0.97 |
| Lithuania | 2021 | 3.10 | 3.00 | -0.10 | 0.10 | 3.27 | +0.17 | 0.17 | 3.13 | +0.03 | 0.03 | 3.63 | +0.53 | 0.53 |
| Lithuania | 2022 | 3.00 | 3.00 | +0.00 | 0.00 | 3.52 | +0.52 | 0.52 | 2.95 | -0.05 | 0.05 | 3.59 | +0.59 | 0.59 |
| Luxembourg | 2018 | 4.30 | 3.20 | -1.10 | 1.10 | 3.25 | -1.05 | 1.05 | 3.24 | -1.06 | 1.06 | 2.71 | -1.59 | 1.59 |
| Luxembourg | 2019 | 4.70 | 3.20 | -1.50 | 1.50 | 3.31 | -1.39 | 1.39 | 3.33 | -1.37 | 1.37 | 2.89 | -1.81 | 1.81 |
| Luxembourg | 2020 | 4.50 | 3.20 | -1.30 | 1.30 | 3.30 | -1.20 | 1.20 | 3.03 | -1.47 | 1.47 | 3.29 | -1.21 | 1.21 |
| Luxembourg | 2021 | 3.10 | 3.20 | +0.10 | 0.10 | 3.08 | -0.02 | 0.02 | 2.55 | -0.55 | 0.55 | 2.62 | -0.48 | 0.48 |
| Luxembourg | 2022 | 3.50 | 3.20 | -0.30 | 0.30 | 3.52 | +0.02 | 0.02 | 2.65 | -0.85 | 0.85 | 3.32 | -0.18 | 0.18 |
| Netherlands | 2018 | 3.50 | 3.60 | +0.10 | 0.10 | 3.45 | -0.05 | 0.05 | 3.52 | +0.02 | 0.02 | 3.11 | -0.39 | 0.39 |
| Netherlands | 2019 | 3.60 | 3.60 | +0.00 | 0.00 | 3.47 | -0.13 | 0.13 | 3.37 | -0.23 | 0.23 | 3.07 | -0.53 | 0.53 |
| Netherlands | 2020 | 3.80 | 3.60 | -0.20 | 0.20 | 3.35 | -0.45 | 0.45 | 3.26 | -0.54 | 0.54 | 3.77 | -0.03 | 0.03 |
| Netherlands | 2021 | 3.30 | 3.60 | +0.30 | 0.30 | 3.30 | +0.00 | 0.00 | 3.19 | -0.11 | 0.11 | 3.32 | +0.02 | 0.02 |
| Netherlands | 2022 | 3.20 | 3.60 | +0.40 | 0.40 | 3.17 | -0.03 | 0.03 | 3.13 | -0.07 | 0.07 | 2.86 | -0.34 | 0.34 |
| Poland | 2018 | 3.80 | 4.00 | +0.20 | 0.20 | 3.96 | +0.16 | 0.16 | 3.73 | -0.07 | 0.07 | 3.47 | -0.33 | 0.33 |
| Poland | 2019 | 3.80 | 4.00 | +0.20 | 0.20 | 3.68 | -0.12 | 0.12 | 3.62 | -0.18 | 0.18 | 2.94 | -0.86 | 0.86 |
| Poland | 2020 | 3.60 | 4.00 | +0.40 | 0.40 | 3.75 | +0.15 | 0.15 | 3.60 | -0.00 | 0.00 | 2.96 | -0.64 | 0.64 |
| Poland | 2021 | 3.90 | 4.00 | +0.10 | 0.10 | 3.68 | -0.22 | 0.22 | 3.46 | -0.44 | 0.44 | 2.81 | -1.09 | 1.09 |
| Poland | 2022 | 3.80 | 4.00 | +0.20 | 0.20 | 3.73 | -0.07 | 0.07 | 3.53 | -0.27 | 0.27 | 2.87 | -0.93 | 0.93 |
| Portugal | 2018 | 3.30 | 2.70 | -0.60 | 0.60 | 3.01 | -0.29 | 0.29 | 2.86 | -0.44 | 0.44 | 2.57 | -0.73 | 0.73 |
| Portugal | 2019 | 2.80 | 2.70 | -0.10 | 0.10 | 3.03 | +0.23 | 0.23 | 2.90 | +0.10 | 0.10 | 2.43 | -0.37 | 0.37 |
| Portugal | 2020 | 2.40 | 2.70 | +0.30 | 0.30 | 3.11 | +0.71 | 0.71 | 3.03 | +0.63 | 0.63 | 2.86 | +0.46 | 0.46 |
| Portugal | 2021 | 2.40 | 2.70 | +0.30 | 0.30 | 2.99 | +0.59 | 0.59 | 2.87 | +0.47 | 0.47 | 3.06 | +0.66 | 0.66 |
| Portugal | 2022 | 2.60 | 2.70 | +0.10 | 0.10 | 2.98 | +0.38 | 0.38 | 2.98 | +0.38 | 0.38 | 2.36 | -0.24 | 0.24 |
| Romania | 2018 | 6.00 | 6.50 | +0.50 | 0.50 | 5.79 | -0.21 | 0.21 | 5.45 | -0.55 | 0.55 | 8.57 | +2.57 | 2.57 |
| Romania | 2019 | 5.80 | 6.50 | +0.70 | 0.70 | 5.64 | -0.16 | 0.16 | 5.42 | -0.38 | 0.38 | 8.66 | +2.86 | 2.86 |
| Romania | 2020 | 5.60 | 6.50 | +0.90 | 0.90 | 5.16 | -0.44 | 0.44 | 4.82 | -0.78 | 0.78 | 8.64 | +3.04 | 3.04 |
| Romania | 2021 | 5.20 | 6.50 | +1.30 | 1.30 | 4.62 | -0.58 | 0.58 | 4.44 | -0.76 | 0.76 | 8.29 | +3.09 | 3.09 |
| Romania | 2022 | 5.70 | 6.50 | +0.80 | 0.80 | 4.51 | -1.19 | 1.19 | 4.22 | -1.48 | 1.48 | 8.08 | +2.38 | 2.38 |
| Slovak Republic | 2018 | 5.00 | 4.50 | -0.50 | 0.50 | 4.33 | -0.67 | 0.67 | 4.51 | -0.49 | 0.49 | 3.79 | -1.21 | 1.21 |
| Slovak Republic | 2019 | 5.10 | 4.50 | -0.60 | 0.60 | 4.12 | -0.98 | 0.98 | 4.41 | -0.69 | 0.69 | 3.71 | -1.39 | 1.39 |
| Slovak Republic | 2020 | 5.10 | 4.50 | -0.60 | 0.60 | 4.12 | -0.98 | 0.98 | 4.29 | -0.81 | 0.81 | 3.76 | -1.34 | 1.34 |
| Slovak Republic | 2021 | 4.90 | 4.50 | -0.40 | 0.40 | 4.04 | -0.86 | 0.86 | 4.23 | -0.67 | 0.67 | 3.54 | -1.36 | 1.36 |
| Slovak Republic | 2022 | 5.40 | 4.50 | -0.90 | 0.90 | 4.06 | -1.34 | 1.34 | 4.30 | -1.10 | 1.10 | 3.62 | -1.78 | 1.78 |
| Slovenia | 2018 | 1.70 | 2.10 | +0.40 | 0.40 | 2.50 | +0.80 | 0.80 | 2.06 | +0.36 | 0.36 | 1.13 | -0.57 | 0.57 |
| Slovenia | 2019 | 2.10 | 2.10 | +0.00 | 0.00 | 2.57 | +0.47 | 0.47 | 2.17 | +0.07 | 0.07 | 1.33 | -0.77 | 0.77 |
| Slovenia | 2020 | 2.20 | 2.10 | -0.10 | 0.10 | 2.52 | +0.32 | 0.32 | 2.14 | -0.06 | 0.06 | 1.79 | -0.41 | 0.41 |
| Slovenia | 2021 | 1.80 | 2.10 | +0.30 | 0.30 | 2.63 | +0.83 | 0.83 | 2.34 | +0.54 | 0.54 | 0.21 | -1.59 | 1.59 |
| Slovenia | 2022 | 2.50 | 2.10 | -0.40 | 0.40 | 2.69 | +0.19 | 0.19 | 2.37 | -0.13 | 0.13 | 0.78 | -1.72 | 1.72 |
| Spain | 2018 | 2.70 | 2.70 | +0.00 | 0.00 | 2.90 | +0.20 | 0.20 | 2.69 | -0.01 | 0.01 | 2.88 | +0.18 | 0.18 |
| Spain | 2019 | 2.60 | 2.70 | +0.10 | 0.10 | 2.89 | +0.29 | 0.29 | 2.57 | -0.03 | 0.03 | 2.60 | -0.00 | 0.00 |
| Spain | 2020 | 2.60 | 2.70 | +0.10 | 0.10 | 2.94 | +0.34 | 0.34 | 2.54 | -0.06 | 0.06 | 3.36 | +0.76 | 0.76 |
| Spain | 2021 | 2.50 | 2.70 | +0.20 | 0.20 | 2.95 | +0.45 | 0.45 | 2.40 | -0.10 | 0.10 | 3.07 | +0.57 | 0.57 |
| Spain | 2022 | 2.60 | 2.70 | +0.10 | 0.10 | 2.94 | +0.34 | 0.34 | 2.40 | -0.20 | 0.20 | 3.12 | +0.52 | 0.52 |
| Sweden | 2018 | 2.00 | 2.40 | +0.40 | 0.40 | 2.65 | +0.65 | 0.65 | 2.52 | +0.52 | 0.52 | 2.30 | +0.30 | 0.30 |
| Sweden | 2019 | 2.10 | 2.40 | +0.30 | 0.30 | 2.67 | +0.57 | 0.57 | 2.75 | +0.65 | 0.65 | 2.43 | +0.33 | 0.33 |
| Sweden | 2020 | 2.40 | 2.40 | +0.00 | 0.00 | 2.61 | +0.21 | 0.21 | 2.34 | -0.06 | 0.06 | 2.64 | +0.24 | 0.24 |
| Sweden | 2021 | 1.80 | 2.40 | +0.60 | 0.60 | 2.65 | +0.85 | 0.85 | 2.40 | +0.60 | 0.60 | 2.46 | +0.66 | 0.66 |
| Sweden | 2022 | 2.20 | 2.40 | +0.20 | 0.20 | 2.78 | +0.58 | 0.58 | 2.14 | -0.06 | 0.06 | 2.34 | +0.14 | 0.14 |

*Note. Columns headed s and |e| are the signed error (predicted minus observed) and absolute error for the model immediately to their left. A positive signed error indicates over-prediction. Predictions and errors for all predictive models, fixed-effects specifications, and benchmarks are provided in Supplementary Data File S1.*

**Supplementary Table S10. Country-level prediction errors (MAE and RMSE) by model for the 2018–2022 test period.**

| **Country** | **Persist MAE** | **Persist RMSE** | **RF+C MAE** | **RF+C RMSE** | **GB+C MAE** | **GB+C RMSE** | **FE(LY) MAE** | **FE(LY) RMSE** |
| --- | --- | --- | --- | --- | --- | --- | --- | --- |
| Austria | 0.220 | 0.272 | 0.453 | 0.509 | 0.269 | 0.283 | 0.406 | 0.464 |
| Belgium | 0.400 | 0.473 | 0.287 | 0.318 | 0.318 | 0.369 | 0.770 | 0.900 |
| Bulgaria | 1.020 | 1.085 | 0.294 | 0.331 | 0.973 | 1.004 | 1.648 | 1.686 |
| Croatia | 0.100 | 0.134 | 0.288 | 0.419 | 0.619 | 0.719 | 0.518 | 0.627 |
| Czechia | 0.300 | 0.344 | 0.678 | 0.716 | 0.567 | 0.605 | 1.688 | 1.711 |
| Denmark | 0.740 | 0.814 | 0.720 | 0.792 | 0.738 | 0.814 | 1.142 | 1.210 |
| Estonia | 0.500 | 0.602 | 1.069 | 1.110 | 0.732 | 0.829 | 0.677 | 0.836 |
| Finland | 0.120 | 0.141 | 0.495 | 0.528 | 0.164 | 0.189 | 0.795 | 0.843 |
| France | 0.160 | 0.179 | 0.304 | 0.390 | 0.362 | 0.465 | 0.239 | 0.261 |
| Germany | 0.160 | 0.179 | 0.274 | 0.281 | 0.205 | 0.227 | 0.807 | 0.874 |
| Greece | 0.200 | 0.276 | 0.293 | 0.376 | 0.219 | 0.336 | 0.286 | 0.316 |
| Hungary | 0.140 | 0.148 | 0.398 | 0.430 | 0.173 | 0.232 | 0.290 | 0.322 |
| Ireland | 0.240 | 0.283 | 0.273 | 0.321 | 0.340 | 0.392 | 2.917 | 3.373 |
| Italy | 0.300 | 0.319 | 0.538 | 0.590 | 0.254 | 0.301 | 0.714 | 0.846 |
| Latvia | 1.060 | 1.141 | 0.674 | 0.712 | 0.508 | 0.583 | 2.577 | 2.608 |
| Lithuania | 0.260 | 0.382 | 0.353 | 0.370 | 0.186 | 0.247 | 0.459 | 0.565 |
| Luxembourg | 0.860 | 1.025 | 0.736 | 0.947 | 1.061 | 1.114 | 1.054 | 1.228 |
| Netherlands | 0.200 | 0.245 | 0.132 | 0.211 | 0.191 | 0.267 | 0.260 | 0.330 |
| Poland | 0.220 | 0.241 | 0.146 | 0.154 | 0.193 | 0.247 | 0.770 | 0.815 |
| Portugal | 0.280 | 0.335 | 0.440 | 0.476 | 0.405 | 0.440 | 0.491 | 0.523 |
| Romania | 0.840 | 0.881 | 0.514 | 0.633 | 0.790 | 0.874 | 2.786 | 2.800 |
| Slovak Republic | 0.600 | 0.623 | 0.965 | 0.990 | 0.752 | 0.779 | 1.418 | 1.431 |
| Slovenia | 0.240 | 0.290 | 0.521 | 0.580 | 0.234 | 0.300 | 1.014 | 1.149 |
| Spain | 0.100 | 0.118 | 0.324 | 0.334 | 0.080 | 0.105 | 0.407 | 0.492 |
| Sweden | 0.300 | 0.361 | 0.572 | 0.608 | 0.379 | 0.461 | 0.334 | 0.377 |

*Note.* Values are mean absolute error (MAE) and root mean squared error (RMSE), expressed as deaths per 1,000 live births, across the five test years for each country and model. Persist = persistence benchmark; RF+C = tuned Random Forest with country information; GB+C = tuned Gradient Boosting with country information; FE(LY) = two-way fixed effects under the last-observed-year-effect prediction rule. Corresponding country-level errors for the remaining predictive specifications are provided in Supplementary Data File S1.
